# Synthesis of Ferulenol by Engineered *Escherichia coli*: Structural Elucidation by Using the In Silico Tools

**DOI:** 10.3390/molecules26206264

**Published:** 2021-10-16

**Authors:** Anuwatchakij Klamrak, Jaran Nabnueangsap, Ploenthip Puthongking, Natsajee Nualkaew

**Affiliations:** 1 Faculty of Pharmaceutical Sciences, Khon Kaen University, Khon Kaen 40002, Thailand; anuwat_kla@yahoo.com (A.K.); pploenthip@kku.ac.th (P.P.); 2 Salaya Central Instrument Facility RSPG, Research Management and Development Division, Office of the President, Mahidol University, Nakhon Pathom 73170, Thailand; jaran.nab@mahidol.ac.th

**Keywords:** *Escherichia* *coli*, biotransformation, 4-hydroxycoumarin, ferulenol, structural annotation, in silico tools

## Abstract

4-Hydroxycoumarin (4HC) has been used as a lead compound for the chemical synthesis of various bioactive substances and drugs. Its prenylated derivatives exhibit potent antibacterial, antitubercular, anticoagulant, and anti-cancer activities. In doing this, *E. coli* BL21(DE3)pLysS strain was engineered as the in vivo prenylation system to produce the farnesyl derivatives of 4HC by coexpressing the genes encoding *Aspergillus terreus* aromatic prenyltransferase (AtaPT) and truncated 1-deoxy-D-xylose 5-phosphate synthase of *Croton stellatopilosus* (CstDXS), where 4HC was the fed precursor. Based on the high-resolution LC-ESI(±)-QTOF-MS/MS with the use of in silico tools (e.g., MetFrag, SIRIUS (version 4.8.2), CSI:FingerID, and CANOPUS), the first major prenylated product (named compound-1) was detected and ultimately elucidated as ferulenol, in which information concerning the correct molecular formula, chemical structure, substructures, and classifications were obtained. The prenylated product (named compound-2) was also detected as the minor product, where this structure proposed to be the isomeric structure of ferulenol formed via the tautomerization. Note that both products were secreted into the culture medium of the recombinant *E. coli* and could be produced without the external supply of prenyl precursors. The results suggested the potential use of this engineered pathway for synthesizing the farnesylated-4HC derivatives, especially ferulenol.

## 1. Introduction

Prenylation is one of the post-structural modifications essential for the increasing biological activities of several natural products [1]. Transferring the prenyl moieties onto the aromatic acceptor molecules often leds to prenylated derivatives with greatly improved therapeutic potency [2]. This process has become a new frontier for developing novel drugs and lead compounds in the pharmaceutical industry, especially for antimicrobial, antioxidant, anti-inflammatory, and anti-cancer agents [3,4,5]. Nevertheless, plant-based production of these valuable products is limited by finite resources, low yields, slow growth rates, seasonal dependency, and rare, or completely absent in some regions [6,7]. Moreover, the chemoenzymatic and total synthesis of the prenylated products is rather difficult, as it is challenged by the structural complexity which requires multiple steps of the uncontrollable regio- and stereoselective prenylation, and expensive starting materials [8,9].

Ferulenol (2), a C-3 farnesylated 4-hydroxycoumarin, is a major constituent in *Ferula communis* (Giant fennel) which possesses many biological activities [5,10]. According to previous studies, this compound exhibits cytotoxicity against various lines of cancer cells including human breast (MCF-7), colon (Caco-2), ovarian (SK-OV-3), and leukemic (HL- 60) in a dose-dependent manner, with the mode of action resembling paclitaxel (taxol) [11,12]. Ferulenol (2) also exerts anti-cancer activity through the downregulation of Bcl2 protein along with upregulation of Bax protein in benzo[a]pyrene-induced lung cancer in a rat model [13]. This indicated ferulenol (2) as a pro-oxidant and chemotherapeutic agent and has been recognized as an interesting lead compound for anti-cancer semi-synthesis [13,14]. In searching for the novel anticoagulant warfarin derivatives, ferulenol (2) exhibits higher activity than the warfarin drug (approximately 22 times) with lower toxicity [15,16]. This compound also possesses antimycobacterial activity against fast-growing *Mycobacterium* species [17]. Although chemical synthesis of this compound could be achieved by the reaction between 4-hydroxycoumarin sodium salt (4HCNa) and all trans-farnesylchloride based on alkylation at the C-3 of coumarin [15], these processes are quite complicated, and the starting precursors are rather expensive.

Engineering microbial cells as the in vivo prenylation system provides several advantages over the two existing methods, as microbes can be grown very fast in the noncomplex medium and can be easily extended to large-scale production [9,18]. More importantly, microbes can supply various lengths of prenyl donors, e.g., dimethylallyl pyrophosphate (DMAPP, C5), geranyl pyrophosphate (GPP, C10), farnesyl pyrophosphate (FPP, C15), and geranylgeranyl pyrophosphate (GGPP, C20), through their inherent isoprenoid pathways [7,19]. *E. coli* solely synthesizes isoprenoids via the 1-deoxy-D-xylulose 5-phosphate (DXP) pathway (also known as non-mevalonate pathway), in which 1-deoxy-D-xylulose 5-phosphate synthase (DXS) is known as the first committing step enzyme that controls the metabolic flux of isoprenoids precursors [20,21,22,23,24]. In wild type *E. coli*, FPP serves as the key branching point in the synthesis of the vital molecules, i.e., heme O, ubiquinone, and peptidoglycan [25,26,27,28]. Consequently, the metabolic flux of this precursor in *E. coli* has been redirected towards taxadiene, carotenoids, and amorpha-4,11-diene through heterologous expression of various terpene synthases [29,30,31,32,33,34]. In the last few years, using microorganisms as the prenylation systems for the production of hybrid molecules containing prenyl moieties has focused mainly on yeasts (e.g., *Saccharomyces cerevisiae* and *Pichia pastoris*) and *Bacillus subtilis* based on the endogenous prenyl donors supplied via either the mevalonate (MVA) pathway or the DXP pathway [35,36,37,38]. However, an attempt at synthesizing ferulenol (2), the product of C-3 farnesylation of 4HC (1), through the genetic manipulation of the DXP pathway in *E. coli* has not been reported yet. Therefore, this gives rise to our interest in establishing a new synthesis pathway for producing this product by using the engineered microbe based on feeding of 4HC (1) to minimize chemical consumption.

Aromatic prenyltransferases (aPTs) are the enzymes that catalyze the regio-selective prenylation of the prenyl groups (so-called prenyl donors), incorporating the aromatic compounds (known as aromatic acceptors) that contain the electron-rich regions through a mechanism comparable to Friedel–Crafts aromatic electrophilic substitution [39,40,41,42]. Nowadays, the genes encoded for aPTs have been isolated from plants, fungi, and bacteria and have been found to exhibit broad substrates with specificity both in terms of aromatic acceptors and prenyl donors, creating a diverse range of prenylated products [1,2,3,43,44,45]. Of those enzymes characterized, *Aspergillus terreus* aromatic prenyltransferase (AtaPT) possesses the broad range substrate specificity towards various types of aromatic acceptors (e.g., coumarins, resveratrol, and naringenin) and prenyl donors, i.e., DMAPP, GPP, FPP, and GGPP, yielding prenylated products with structural diversity [46]. This enzyme also differs from formerly characterized aPTs, as it can generate mono-, di-, and/or triprenylated products via C-C- and/or C-O-bonded prenylation. Since 4-hydroxycoumarin (4HC) (1) contains the two promising regions for electrophilic alkylation, including the C3 and the oxygen atom at C4 positions [15,47], it was proposed to be utilized by AtaPT due to the unique catalytic activity of this enzyme. In wild-type *E. coli*, its native isoprenoid precursors are always insufficient for pathway engineering purposes, therefore driving metabolic fluxes via overexpression of the rate-limiting step enzymes in the DXP pathway, e.g., DXS, 1-deoxy-D-xylulose 5-phosphate reductoisomerase (DXR), and isopentenyl pyrophosphate isomerase (IDI) are required [19,20,21,22,23,24].. According to the previous findings, transcriptional profiling analysis revealed a positive correlation between CsDXS gene expression and plaunotol (acyclic diterpene alcohol) content in the young leaves of *Croton stellatopilosus*, and this enzyme has been suggested to control the metabolic flux of isoprenoid precursors in that plant [48]. Therefore, the truncated CsDXS (namely CstDXS) was chosen to co-express with the AtaPT in *E. coli* BL21(DE3)pLysS to establish a new synthetic route for the in vivo formation ferulenol (2), in which 4HC (1) was only a fed precursor.

**Figure 1:**
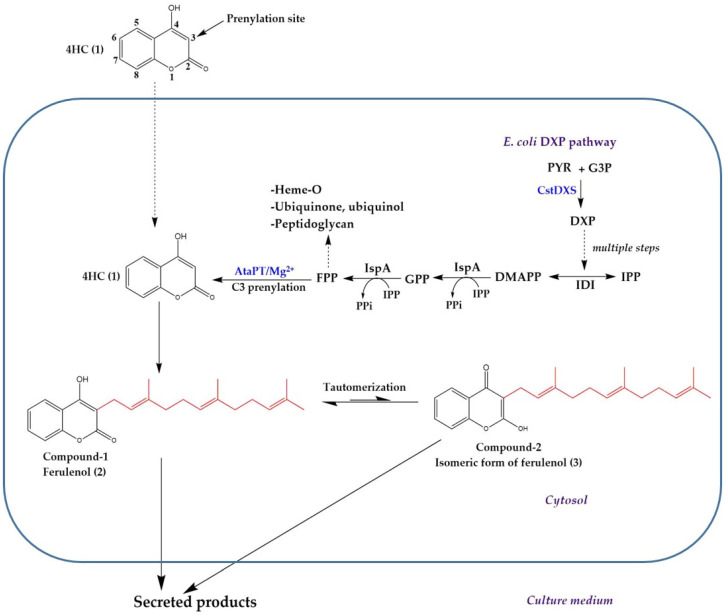
Proposed biosynthetic pathway of ferulenol (2) and its isomeric structure (3) reconstructed in *E. coli* BL21(DE3)pLysS carrying pCDFDuet-AtaPT-CstDXS, where 4HC (1) was the fed precursor. Note that both products can be formed without any supply of prenyl donors (i.e., FPP) and secreted into the culture medium. PYR = pyruvate; G3P = glyceraldehyde 3-phosphate; IPP = isopentenyl pyrophosphate; DMAPP = dimethylallyl pyrophosphate; GPP = geranyl pyrophosphate; FPP = farnesyl pyrophosphate; IDI = isopentenyl pyrophosphate isomerase; IspA = geranyl pyrophosphate synthase and farnesyl pyrophosphate synthase.

In this study, *E. coli* was used as the bioconversion system of the newly designed pathway leading to the formation of farnesylated-4HC analogs, which includes two steps (Figure 1). The CstDXS was overexpressed for driving metabolic flux of isoprenoid precursors (e.g., GPP, FPP) in the DXP pathway [47]. Second, prenylation of those prenyls to the core structure of 4HC (1) through the catalytic function of AtaPT produced its corresponded products (2 and 3) [46]. This process requires magnesium ion (Mg^2^^+^) as a cofactor to facilitate the formation of an electrophilic prenyl carbocation, which subsequently binds to 4HC (1) via the Friedel-Crafts reaction [46].

The structure elucidation of the target compounds by NMR techniques has been limited to the study of metabolic engineering and metabolomics, which deals with trace-level metabolites [49]. Therefore, high-resolution LC-ESI-QTOF-MS/MS has played a crucial role instead through its high sensitivity and can distinguish between the closed atomic mass numbers [49,50,51]. The experimental mass spectra can be interpreted into the chemical structures based on the in silico prediction tools by matching against the in silico -fragmented spectra of compounds in databases (i.e., PubChem, MetaCyc) [51,52,53]. In this study, a variety of the in-silico tools were chosen for the structurally assisted identification of the prenylated-4HC derivatives obtained from the bioconversion of 4HC (1) by the clones carrying pCDFDuet-AtaPT-CstDXS to verify the product formation.

Here, we report the newly artificial pathway for synthesizing ferulenol (2) established in the *E. coli* BL21(DE3)pLysS strain, where the chemical structures of this product are entirely elucidated by using in silico tools, included MetFrag [52], SIRIUS [54], CSI:FingerID web service [55], and CANOPUS [56]. The experimental mass spectra for the putative ferulenol (2) are further confirmed by the alignment to the mass spectra of the authentic ferulenol (2) established using the same LC-MS/MS condition, along with those established by Fourel and colleagues [57]. The putative mass peak corresponds to farnesylated derivative of 2-hydroxy-4-chromenone, which has been proposed to exist via the tautomerization of ferulenol (2) and is detected in this study. The results can be used to further establish the *E. coli* system as the microbial cell factory for producing the farnesylated-4HC derivatives to serve drug discovery purposes.

## 2. Results

### 2.1. Construction of Plasmid pCDFDuet-AtaPT-CstDXS

**Figure 2:**
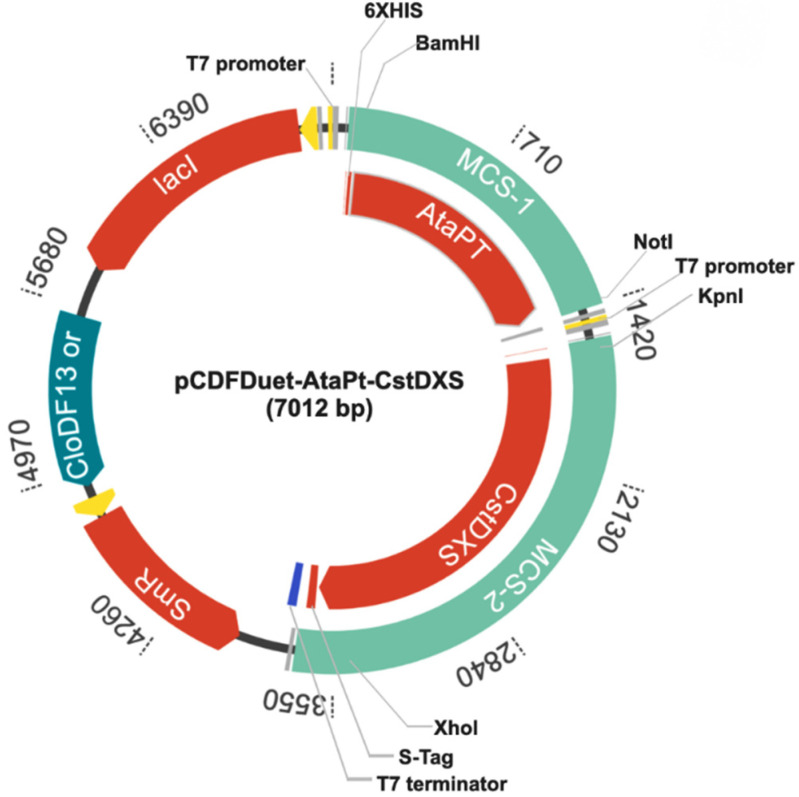
Map of recombinant plasmid pCDFDuet-AtaPT-CstDXS.

Engineering *E. coli* as the in vivo prenylation system requires at least two steps: (1) overexpression of CstDXS as the rate-limiting step enzyme in the DXP pathway to increase the available pool of prenyl-donors; and (2) transferring the prenyl-donors into the core aromatic acceptor by the catalytic function of AtaPT. Therefore, the truncated DXS (CstDXS: GenBank accession no. AB354578.1) was used for enhancing the flux of isoprenoid precursors in *E. coli*. The gene encoding for AtaPT (GenBank accession no. KP893683) was chosen for incorporating the prenyl donors into the 4HC core structure. Construction of pCDFDuet-AtaPT-CstDXS was achieved via stepwise incorporation of AtaPT, followed by CstDXS into the multiple cloning site 1 (MCS-1) and MCS-2 of pCDFDuet-1 vector, respectively. DNA sequencing confirmed that both genes were inserted into the correct regions of the pCDFDuet-1 vector with no frame-shifted insertion. Each gene was independently controlled by their T7 promotor, and gene expression was driven by adding IPTG into the culture (Figures S1 and S2). The 5’ end of AtaPT in the MCS-1 (*BamH*I/*Not*I) was joined with His-tag, and the 3′ end of CstDXS was fused with the S-tag. The map created using GenScript (https://www.genscript.com/gensmart-design/# (accessed on 26 September 2021)) is depicted in Figure 2.

### 2.2. Identification of Prenylated 4HC Derivative Using LC-ESI(-)-QTOF-MS/MS 

**Figure 3:**
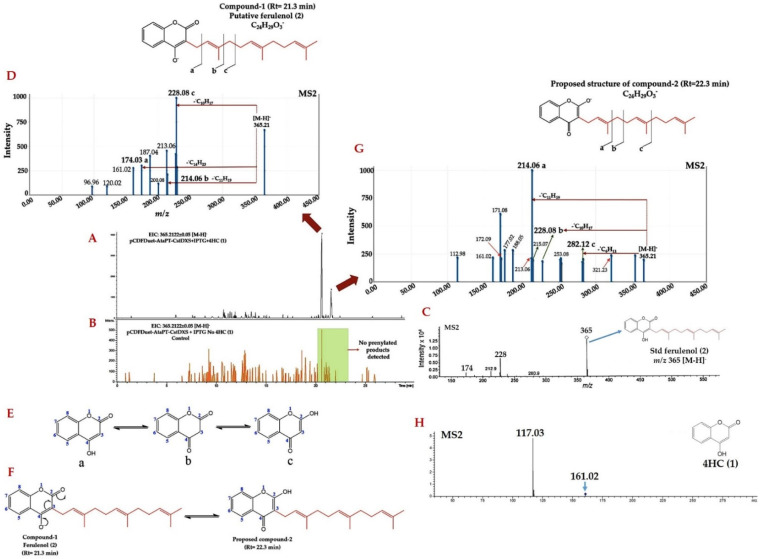
Identification of prenylated products using negative-ion mode LC-ESI-QTOF-MS/MS. (**A**) The EIC for molecular ion with *m/z* 365.2122 ± 0.0500 [M − H]^−^ detected from the medium extract of clones carrying pCDFDuet-AtaPT-CstDXS grown in the presence of fed 4HC (1). (**B**) The EIC for molecular ion with *m/z* 365.2122 ± 0.0500 [M − H]^−^ of the clones carrying pCDFDuet-AtaPT-CstDXS grown in the absence of 4HC (1) (control group). (**C**) The MS/MS spectrum of authentic ferulenol (2) (1 μg/mL) established by Fourel et al. [57] (**D**) The MS/MS spectrum of compound-1 (*m/z* 365.21; Rt = 21.3 min). (**E**) The tautomerization of 4HC (1) reported by Abdou et al. [47]. (**F**) The proposed mechanism underlying the formation of compound-2 (3), which proceeded via the tautomerization of ferulenol (2) (compound-1). (**G**) The MS/MS spectrum of compound-2 (*m/z* 365.21; Rt = 22.3 min). (**H**) The MS/MS spectrum of 4HC (1) detected from the culture medium of clones harboring pCDFDuet-AtaPT-CstDXS.

The high-resolution LC-ESI-QTOF-MS/MS was used for the identification of the prenylated 4HC derivative produced by clones carrying pCDFDuet-AtaPT-CstDXS. The in vivo formation for the peak corresponding to the 4HC containing farnesyl moiety was detected on the extracted ion chromatogram (EIC) at the calculated *m/z* 366.211670 [M − H]^−^, in which the product was tentatively confirmed to be “ferulenol (2)” by comparing the MS/MS fragmentation profile against the authentic ferulenol (2) established by the previous work [57]. The results showed that there were two mass peaks (namely compound-1 and -2) which exhibited the identical *m/z* 365.21 eluted at the RT 21.3 min and 22.3 min, respectively (Figure 3A). No product formation was observed in the culture medium clones harboring pCDFDuet-AtaPT-CstDXS grown without supplementing 4HC (1) in the culture (Figure 3B). These products were therefore presumably farnesylated-4HC, where the farnesyl moiety was incorporated on the C3 position of 4HC (1) via C-C-bonded formation. Based on the direct comparison against previous mass spectra, only the compound-1 highly resembled the MS/MS profile to that of ferulenol (2) reported by Fourel et al. [57], which was characterized by the ion peak *m/z* 365 [M − H]^−^, 228 and 174 (Figure 3C). In the MS/MS spectrum of this product, the presence of product ions with *m/z* 228.08 (base peak), 214.06, and 174.03 illustrated the partial losses of prenyl side chain attached to the C3 position of 4HC (1), which was the core structure of this prenylated product (Figure 3D). This product (compound-1) is therefore believed to be ferulenol (2), where the obtained mass data (Figure S4) were further eluicidated by many aspects of in silico tools, including MetFrag web service, SIRIUS, CSI:Finger ID web service, and CANOPUS to entirely support the structural elucidation of this prenylated product. Abdou et al. [47] revealed that 4HC (1) is able to exist in tautomeric forms including 4-hydroxy-2-chromenone (a), 2,4-chromadione (b), and 2-hydroxy-4-chromenone (c) (Figure 3E). The compound-2 (*m/z* 365.21; Rt = 22.3 min) was thus presumably the isomeric form of ferulenol (2) proceeded via the tautomerization, where the 2-hydroxy-4-chromenone (c) served as the aromatic core structure (Figure 3F). The presence of fragmented ions with *m/z* 214.06 (base peak), 228.08, and 282.12 in the resulting MS/MS spectrum also indicated the partial losses of prenyl moiety that attached to the C3 position of this prenylated product (Figure 3G). Note that 4HC (1) as the fed substrate was also detected in the culture medium of the clones carrying pCDFDuet-AtaPT-CstDXS (Figure 3F).

### 2.3. Structural Annotation of Compound-1 by Using MetFrag Web Service 

**Figure 4:**
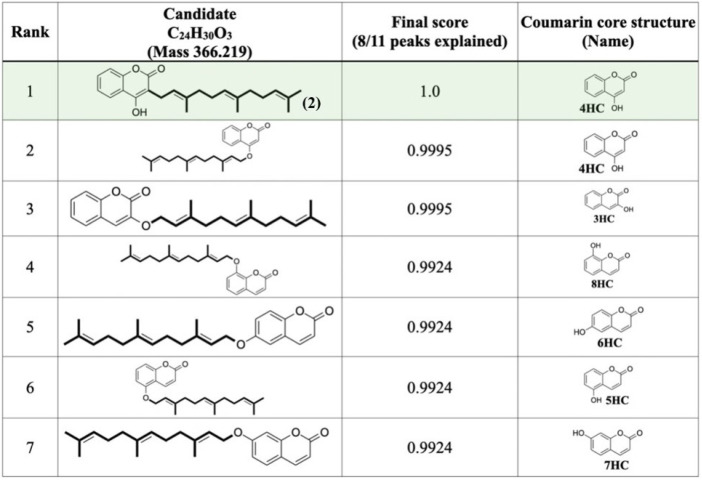
Structural annotation of compound-1 (*m/z* 365.21 [M − H]^−^; Rt = 21.3 min) using MetFrag web service, where the query subject was best annotated as ferulenol (2) among 2267 structures retrieved from the PubChem database.

The chemical structure of compound-1 was further annotated by using MetFrag web service, the in silico tool designed to elucidate chemical structure of query subjects from the experimental mass spectra [52]. Among the 2267 candidates retrieved from the PubChem database, compound-1 was best annotated as ferulenol (2), (compound CID: 54679300), with the highest score of 1.0 in which 8/11 peaks were matched with those of the in silico-generated fragmented ions of the candidated ferulenol (2) deposited in the PubChem database (Figure 4). We hence suggested that compound-1 is likely to be ferulenol (2), rather than the other prenylated 4HC derivatives exhibiting the identical mass value with *m/z* of 365.21 [M − H]^−^.

### 2.4. Computationally Assisted Identification of the Prenylated Product by Using SIRIUS (Version 4.8.2)

According to the user manual, SIRIUS requires high mass accuracy in which the ppm error is less than 20 ppm before conducting the annotation processes for the most reliable results [55]. The observed molecular ion *m/z* 365.21 [M − H]^−^ was thus estimated by using the mass error calculation tool from the web service (https://warwick.ac.uk/fac/sci/chemistry/research/barrow/barrowgroup/calculators/mass_errors/ (accessed on 17 June 2021)), by comparing against its theoretical *m/z* (365.212218 [M − H]^−^), and showed an error of 8.762029 ppm, permitting the elucidation of the raw mass data by using this tool.

**Figure 5:**
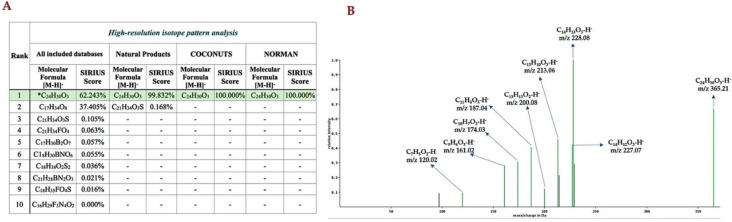
Molecular formula annotation of the prenylated 4HC analog produced by clones carrying pCDFDuet-AtaPT-CstDXS using SIRIUS (version 4.8.2). (**A**) High-resolution isotope pattern analysis reveals the molecular formula of query subject is best annotated as C_24_H_30_O_3_. (**B**) The mass spectra is annotated to match with the local databases of SIRIUS (indicated by the green spectrum with corresponding molecular formula), while the spectra with no annotations are considered to be noise peaks (indicated by the black spectrum).

SIRIUS utilizes the high-resolution isotopic pattern analysis to locally annotate the correct molecular formula based on the experimentally acquired MS/MS data of the query subjects. Among the potential ten elemental formulas retrieved from the PubChem database, our query subject was annotated as C_24_H_30_O_3_ with the highest Sirius score of 62.243% (Figure 5A), which was identical to the native elemental formula of ferulenol (2) (C_24_H_30_O_3_) deposited in the PubChem database (https://pubchem.ncbi.nlm.nih.gov/compound/Ferulenol (accessed on 17 June 2021)). SIRIUS also offers a refined search option to explore the query subjects against biological databases, e.g., Natural Products, Collection of Natural Products (COCONUTS), and NORMAN databases to specify and narrow natural molecules at user-defined cut-offs. By searching the possible structure against the aforementioned databases, our query metabolite (compound-1) was annotated as C_24_H_30_O_3_ with a score that greatly improved to 100% (Figure 5A). The annotated MS/MS spectra locally computed by the SIRIUS tool revealed that 8 of 11 peaks (indicated in the green spectra) matched with their local database (Figure 5B). The result was also consistent with those fragmentation spectra predicted by MetFrag, where 8 out of 11 peaks matched with the in silico fragmented ions of the candidate ferulenol (2) in the PubChem database (Figure 4).

### 2.5. Structural Annotation and Compound Classification of Compound-1 by Using CSI:FIngerID and CANOPUS 

**Figure 6:**
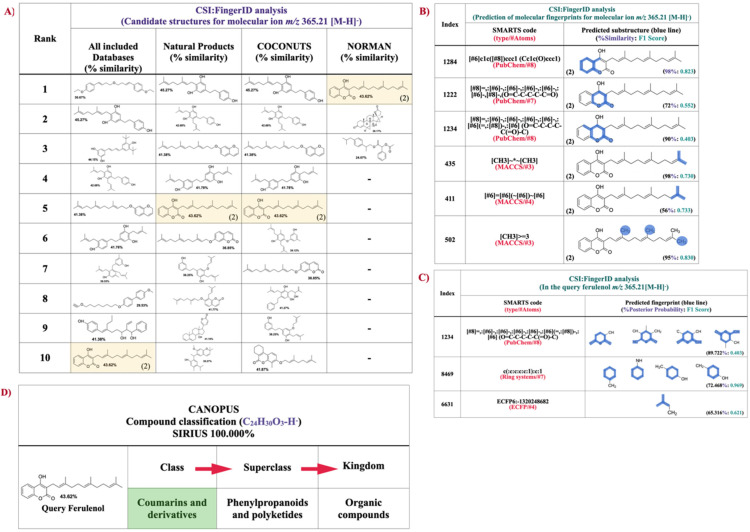
Structural annotation and classifications for the query ferulenol (2) (*m/z* 365.21 [M − H]^−^) synthesized by the recombinant *E. coli* carrying pCDFDuet-AtaPT-CstDXS based on CSI:FingerID and CANOPUS integrated in the SIRIUS (version 4.8.2). (**A**) In all included databases, ferulenol (2) as the target metabolite was ranked as the tenth potential candidate (highlighted in yellow) for the prenylated 4HC analog *m/z* 365.21 [M − H]^−^, meanwhile, the rank of this product significantly increased towards the fifth- and top-ranked candidates when structural identification was performed against the biological databases included Natural Products, COCONUTS, and NORMAN. (**B**) The examples of substructures (molecular fingerprints) predicted to exist in the query subject, including those belonging to the 4HC (1) core structure (No. 1284, 1222, and 1234), and the farnesyl moiety (No. 435, 411, and 502). (**C**) The examples of substructures belonging to ferulenol (2) found to exist in the training structures of the SIRIUS tool. (**D**) Based on the experimentally observed MS/MS data, CANOPUS showing the query compound (C_24_H_30_O_3_ (SIRIUS score 100%), *m/z* 365.21 [M − H]^−^) was categorized as coumarins and derivatives, describing the polycyclic aromatic compounds that contains a 1-benzopyran moiety with a ketone group at the C2 carbon atom (1-benzopyran-2-one).

SIRIUS has recently been integrated with CSI: FingerID web service to identify the chemical structure of the query subjects [55]. In this step, the annotated mass spectrum (Figure 5B) was compared against several compounds in the chosen molecular structure databases (e.g., PubChem, MeSH, and COCONUTS). Of more than 100 possible structures retrieved from all databases and locally predicted by the tool, ferulenol (2) as the target product was ranked as the tenth candidate structure with a percentage similarity of 43.62% (Figure 6A). Since ferulenol (2) is a natural product exclusively produced by *F. communis*, we narrowed the scope of the structural elucidation by using Natural Products and COCONUTS databases and found the rank of the candidate was substantially improved from tenth to fifth (Figure 6A). By searching against the structural compounds from the NORMAN database, our prenylated 4HC analog (compound-1) was perfectly matched to that of ferulenol (2) as the top ranked candidate (Figure 6A). Besides, CSI:FingerID can provide the crucial information of so-called “molecular fingerprints” to verify various substructures that can be found in the query subjects (Figure 6B). In this instance, several molecular fingerprints belonging to ferulenol (2) were predicted to be present in this prenylated product (*m/z* 365.21 [M − H]^−^). For example, substructures encoded by a SMARTS string “[#6]c1c([#8])ccc1 (Cc1c(O)ccc1)” with a score of 98% (F1 = 0.823) correspond to the benzene ring attached to the pyrone ring of 4HC (1). The basis pyrone ring of 4HC encoded by [#8]=,:[#6]-,:[#6]:[#6]-,:[#8](O=C-C:C-O) possessed 85% similarity (F1 = 0.815) and was verified to be present in same candidate structure. Biosynthetically, they were all obtained from the 4HC (1) fed in the culture medium of clones bearing pCDFDuet-AtaPT-CsTDXS. Equally important, there were several substructures belonging to the farnesyl moiety which originated from the DXP pathway, predicted to be present in the same candidate structure. Several substructures representing the basic benzene and pyrone rings along with the isoprene building block belonging to the query ferulenol (2) were predicted to be present in the trained structures of SIRIUS tool (Figure 6C). SIRIUS has also been developed to connect with CANOPUS (class assignment and ontology prediction using mass spectrometry) for logical classification of unknown metabolites based on the high-resolution MS/MS data [56]. According to the molecular properties annotated by CSI:FingerID web service, our query prenylated product *m/z* 365.21 [M − H]^−^ was systematically classified as coumarins and derivatives (class), where phenylpropanoids/polyketides and organic compounds served as the superclass and kingdom, respectively (Figure 6D). CANOPUS also provides alternative classes of the query subjects. In this case, the putative ferulenol (2) was mainly classified as a coumarin derivative, but aromatic monoterpenoids, benzenoids, and lactones were recognized as the alternative classes of this product. Based on the data acquired from the direct comparison against previously established mass spectra [56] along with the molecular formula and structural elucidation by means of the in silico tools included, MetFrag, SIRIUS, CSI:FingerID, and CANOPUS strongly supported that compound-1 was ferulenol (2), which is the product of C3-farnesylation of 4HC (1).

### 2.6. Identification of Farnesylated-4HC Derivatives by Using LC-QTOF-MS/MS in Positive ESI Mode

**Figure 7:**
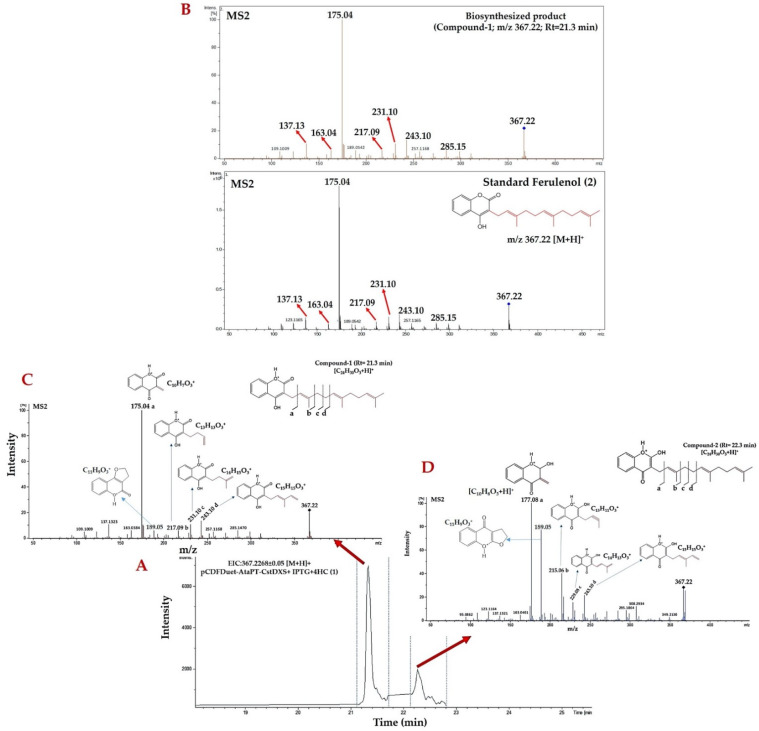
Identification of prenylated products produced by clones carrying pCDFDuet-AtaPT-CstDXS based on the high-resolution LC-ESI-QTOF-MS/MS in the positive-ion mode. (**A**) The EIC for the molecular ion *m/z* 367.2268 ± 0.05 [M + H]^+^ showing two reaction products exhibited the equivalent *m/z* 367.22 (named compound-1 and 2) eluted from the HPLC colume at the retention times of 21.3 and 22.3 min, respectively. (**B**) The direct comparison between the MS/MS spectrum of compound-1 (*m/z* 367.22) and the authentic ferulenol (2) (*m/z* 367.22), which is characterized by the product ions with *m/z* 137.13, 163.04, 175.04, 217.09, 231.10, 243.10, and 285.15, respectively. (**C**) The MS/MS spectrum of compound-1 showing the partial losses of prenyl side chain from the 4HC core structure (1). (**D**) The MS/MS spectrum of compound-2 (proposed structure) signifying the partial elimination of prenyl moiety from the 2-hydroxy-4-chromenone, a core structure of the prenylated product.

The high-resolution LC-QTOF-MS/MS in positive ion mode was thus implemented to corroborate both prenylated products based on monitoring of the molecular ion *m/z* 367.227320 [M + H]^+^ computed by the web tool (https://www.sisweb.com/referenc/tools/exactmass.htm?formula=C24H31O3 (accessed on 7 August 2021)). In the extracted ion chromatogram (EIC) of the medium extract of clones carrying pCDFDuet-AtaPT-CstDXS, there were two extracted ions (namely compound-1 and compound-2) eluted at the retention times of 21.3 and 22.3 min that exhibited identical *m/z* 367.22 [M + H]^+^, where they presumably farnesylated derivatives of 4HC (Figure 7A). The resulting MS/MS spectrum of compound-1 (*m/z* 367.22; Rt = 21.3 min) was clearly identical to those belonging to the authentic ferulenol (2) established under the same LC-MS/MS condition (Figure 7B). In the MS/MS spectrum of this prenylated product, the presence of proposed substructures at *m/z* 175.04 (base peak), 217.08, 231.10, and 243.10 illustrated the partial losses of the prenyl side chain specifically attached to a C3 position of the 4HC core structure (1) (Figure 7C). The obtained evidence leds us to suggest that the compound-1 (*m/z* 367.22 [M + H]^+^; Rt 21.3 min) was indeed ferulenol (2), a product derived from C3-farnesylation of 4HC (1). Hence, the compound-2 (*m/z* 367.22 [M + H]^+^; Rt = 22.3 min) was presumably the tautomeric structure of ferulenol (2), where the 2-hydroxy-4-chromenone (c) served as the core structure (Figure 3E). In the MS/MS spectra along with proposed structures of this product, the existing product ions at *m/z* 177.08 (base peak), 215.06, 229.08, and 243.10 signify the partial elimination of prenyl moiety that incorporated the C3 region of 2-hydroxy-4-chromenone (Figure 7D). The existing of signals at *m/z* 189.05, which were presented in the MS/MS spectrum of both products as shown in Figure 7C,D, were presumably obtained from the prenyl moieties which were adjacent to the core structure of ferulenol (2) and its isomeric structure (3). Based on the acquired evidence, compound-2 was tentatively defined as the isomeric structure of ferulenol (2) which was formed via “tautomerization”. Further elucidation (e.g., NMR) is required to verify the tentative confirmation of this prenylated product. The proposed reaction mechanisms illustrating the various chemical losses present in the MS/MS spectrum of compound-1 and compound-2 are shown in Figures S5 and S6. The postulated mechanisms underlying the formation of ions *m/z* 189.05 of compound-1 and compound-2 were shown in the Supplementary Materials (Figures S7 and S8).

## 3. Discussion

*E. coli* has the capability to supply various isoprenoid precursors through the native DXP pathway [19,20,21,22,23,24]. In the past few decades, this microorganism has been engineered to produce various bioactive terpenoids and valuable precursors such as limonene, taxadiene (taxol precursor), amorphadiene (artemisinin precursor), and carotenoids (e.g., lycopene, astaxanthin, carotenoids) by the heterologous expression of terpene synthases with the rate-limiting step enzymes in the DXP pathway [29,30,31,32,33,34]. However, few studies have focused on the use of this microorganism as the in vivo prenylation system of aromatic natural products. In wild-type *E. coli*, FPP is involved in the biosynthesis of various molecules (e.g., ubiquinone and peptidoglycan) and its level is tightly maintained, as it is essential for bacterial growth and viability [25,26,27,28]. A previous study also demonstrated that the deletion of genes encoded for FPP synthase (IspA) resulted in the observed growth retardation in the mutant strains of *E. coli* [25]. We hence speculated that FPP might become more readily available for the in vivo prenylation of 4HC (1) for ferulenol (2) than others (e.g., DMAPP, GPP, and GGPP). However, the native isoprenoid precursors of *E. coli* are always insufficient for metabolic engineering purposes, and the enhanced metabolic fluxes via overexpression of the rate-limiting step enzymes in the DXP pathway (e.g., DXS, DXR, and IDI) are required [7,19,20,21,22,23,24]. The transcriptional profile analysis in the young leaves of *C. stellatopilosus* revealed a positive association between CsDXS gene expression and plaunotol (acyclic diterpene alcohol) content, and this enzyme was hence suggested as one of the rate-limiting steps in the DXP pathway controlling the flux of isoprenoid precursors in the plant [48]. Since the active form of CsDXS could be achieved after the removal of the signaling peptide region [30,48], the truncated form of this enzyme, CstDXS, was used in this study to drive the metabolic flux of the DXP pathway in the *E. coli* BL21(DE3)pLysS strain. We demonstrated that *E. coli* BL21(DE3)pLysS harboring pCDFDuet-AtaPT-CstDXS is capable of producing the putative mass peak corresponding to ferulenol (2) from the fed 4HC (1) without relying on the external supply of prenyl-donors (e.g., DMAPP, GPP, and FPP). The synthesized product was found to be excreted into the culture medium. This means the step of breaking the cell during the downstream processes is not necessary. Furthermore, this expression system did not require the use of high-priced precursors. These reflected the potential economic system of the prenylated 4HC derivatives production by using recombinant *E. coli*. Besides, our results extend the findings of Chen et al. [46] in that AtaPT can utilize various substrates by showing that 4HC (1) could also be recognized as the aromatic acceptor, even though it has never been reported that this compound acted as the substrate of AtaPT.

Although NMR elucidation is required to verify the structures of the resulting prenylated product (compound-1), this technique is restricted to researchers in the fields of metabolic engineering and metabolomics, which have to deal with trace-level metabolites [49,50,51]. The structural elucidation of compound-1 was thus based on the use of in silico tools designed to annotate the structures of the query metabolites using the experimental mass spectra (MS2) (Figure S4). Having been confirmed to be highly consistent with the unique MS/MS fragmentation profile of ferulenol (2) from the previous work [56], MetFrag analysis clearly showed that the compound-1 was best annotated as “ferulenol (2)” out of the 2266 candidates presented from PubChem database. However, information regarding the molecular formula, chemical structure, substructures, and classifications of this product are still needed. SIRIUS is one of the in silico tools developed to unravel various chemical features hidden in the MS/MS data of the query subjects [52]. This tool has typically been used in the field of metabolomics and has been shown to be helpful in the field of metabolic engineering, in which it was used to identify 2,4,6-trihydroxybenzophenone produced by the engineered *E. coli* [58]. The results from SIRIUS showed that our query product (compound-1) possessed the neutral molecular formula C_24_H_30_O_3_, which corresponded to that of ferulenol (2) deposited in the PubChem database (https://pubchem.ncbi.nlm.nih.gov/compound/Ferulenol (accessed on 26 September 2021)). SIRIUS also locally computed the relevant MS/MS spectra and fragmentation pathway which might be involved in the fragmentation process of compound-1 in which eight mass peaks were explained, meanwhile, the rest of the three peaks were considered as the noises. Based on the eight mass peaks explained by SIRIUS, CSI:FingerID analysis suggested that the compound-1 was annotated as ferulenol (2) as the tenth-ranked candidate after searching against all databases provided by the SIRIUS tool. Although the expected ferulenol (2) was not perfectly categorized as the first candidate for the compound-1, it might be speculated that CSI:FingerID integrated in SIRUS exhibited extremely good performance in the case of the independent MS/MS being trained and deposited in the training data, and the rate of correct identification was substantially decreased when the trained data were removed from this tool [54]. This can be confirmed by the fact that the MS/MS data for the authentic ferulenol (2) (encoded by InChI=1S/C24H30O3/c1-17(2)9-7-10-18(3)11-8-12-19(4)15-16-21-23(25)20-13-5-6-14-22(20)27-24(21)26/h5-6,9,11,13-15,25H,7-8,10,12,16H2,1-4H3/b18-11+,19-15+) has not yet been trained in the negative mode mass data of the SIRIUS tool (https://www.csi-fingerid.uni-jena.de/v1.6.0/api/fingerid/trainingstructures?predictor=2 (accessed on 23 June 2021)). Based on the LC-MS search (for the molecular ion *m/z* 365.21 [M − H]^−^), it might also be possible to exclude the rest of candidates (ranked first to ninth retrieved from all included databases, and ranked first to fifth from the Natural Products and COCONUTS databases) as incorrect structures since they are not natural occurring in *E. coli*, and there was only 3b-allotetrahydrocortisol showing the proximal *m/z* 365.23 [M − H]^−^ found in the *E. coli* metabolomics database (ECMDB) (https://ecmdb.ca/spectra/ms/search (accessed on 23 June 2021)). This indicated that only ferulenol (2) as the product of pathway engineering could be accepted as the correct structure. In the case of the query metabolites acquired from the biological samples, SIRIUS also provides the biological databases as the choices to narrow the scope of natural molecules. When MS/MS spectra of compound-1 were annotated against biological databases, e.g., Natural Products, COCONUTS, and NORMAN, the rank annotated as “ferulenol (2)” was greatly improved as the fifth and first candidate, respectively. Based on the substructures predicted by CSI:Finger ID and CANOPUS, it subsequently provided the vital information that the compound-1 was classified as coumarins and derivatives.

According to Abdou and colleaques [47], the 4HC (1) can exist in three different tautomeric structures: 4-hydroxy-2-chromenone (a), 2,4-chromadione (b), and 2-hydroxy-4-chromenone (c) (Figure 3E). We hence postulated that the compound-2 (eluted from the HPLC column at 22.3 min), obtained from the negative- and positive-ion mode analyses, is presumably the tautomeric form of ferulenol (2). The obtained MS/MS spectra (Figure 3G and Figure 7D) were further elucidated to gain insight into the structural information of this prenylated product. In the negative ion mode analyses, the three chemical losses explained the daughter ions at *m/z* 214.06, 228.08, 282.12, signifying the prenyl side chain was partially removed from the 2-hydroxy-4-chromenone (c), the core structure of this prenylated product. A similar trend was observed in the positive ion mode analyses, where the partial loss of prenyl side chain attaching to the 2-hydroxy-4-chromenone could be characterized via the signals with *m/z* 177.08, 215.06, 229.08, and 243.10, respectively. The existence of product ions at *m/z* 189.05 [M + H]^+^ in the MS/MS spectrum of both prenylated products (Figure 7C,D) were likely to be the outcome of non-enantioselective epoxidation of the double bond present in the farnesyl side chain of ferulenol (2), as recently described by Cortés et al. [59]. Based on the obtained evidence, the compound-2 (3) was interpreted as the farnesylated derivative of 2-hydroxy-4-chromenone, however further elucidation (e.g., NMR) is required to fully support a chemical point of view.

The availability of prenyl precursors influences the patterns of isoprenoid-derived natural products produced by the bacterial systems [60]. Here, ferulenol (2) and its proposed isomeric structure (3) were exclusively detected as the predominant products produced by *E. coli*-carried pCDFDuet-AtaPT-CstDXS. Although AtaPT exhibits remarkable substrate promiscuity towards a variety of prenyl-donors such as DMAPP, GPP, FPP, and GGPP [46], the presence of two farnesylated-4HC analogs (compounds-1 and -2) clearly supports our hypothesis that the FPP accumulated inside the bacterial cells is more easily accessible for synthesizing the two prenylated products rather than the others (DMAPP, GPP, and GGPP). Our result was also consistent with the previous finding, where the farnesylated-menadione was the major product of the whole-cell catalysis by *P. pastoris*, harboring the gene encoding for aromatic prenyltransferase (NovQ) [37]. Studies have shown that overexpression of multiple rate-limiting enzymes in the DXP pathway, including 1-deoxy-D-xylulose 5-phosphate reductoisomerase (DXR), isopentenyl pyrophosphate isomerase (IDI), along with FPP synthase (e.g., IspA), causes the substantially increased production of terpenoids in their engineered microbes [23,24,25]. Furthermore, 4HC (1) as the fed precursor is unlikely and unsustainable from an economic standpoint, as a considerable amount of this expensive substrate must be supplied to the bacterial culture to make its desired prenylated analogs. This strategy might be impracticable for industrially scaled applications, where the newly designed strains capable of de novo manufacture of the two farnesylated-4HC analogs or the usage of the lower cost precursors (e.g., sodium salicylate) should be established. Previous research has shown that *E. coli* can be engineered to produce 4HC (1) via the inherent chorismate pathway, which is accomplished through a series of reactions catalyzed by isochorismate synthase (ICS), isochorismate pyruvate-lyase (IPL), salicylate-CoA ligase (SCL), and biphenyl synthase (BIS) [61]. Based on the broad substrate specificity of benzoate-CoA ligase (BadA) and benzophenone synthase (GmBPS), our group demonstrated that *E. coli* BL21(DE3)pLysS carrying pETDuet-BadA-GmBPS was able to synthesize 4HC (1) from the fed salicylate (sodium salt) as well (data unpublished). By considering these advantages, further establishments of *E. coli* systems capable of synthesizing 4HC (1) from the inexpensive precursors (e.g., glucose, sodium salicylate) to act as the ATaPT’s substrate along with enhancing the flux of isoprenoid precursors would be beneficial for large-scale production of the two farnesylated-4HC analogs reported herein.

Although we demonstrated that the *E. coli* BL21(DE3)pLysS strain could be engineered to produce ferulenol (2) and its isomeric structure (3), further optimizations are needed to improve the yields of final product, which seems to be limited by the supply of isoprenoid precursors [7,8,9]. Several studies have demonstrated that overexpression of the multiple enzymes regulating the flux of isoprenoid precursors led to greatly improved terpenoid-derived natural products in *E. coli* [19,20,21,22,23,24]. Future pathway engineering via overexpression of the other rate-controlling steps in the DXP pathway, such as DXR, IDI, and farnesyl pyrophosphate synthase (FPPS), will be carried out to improve the yields of the two prenylated-4HC derivatives reported herein. Since a large amount of 4HC (1) was also found to remain in the culture medium, optimizing substrate consumptions, i.e., a time course production is needed to enhance the yield of those prenyl derivatives. There are several factors affect the heterologous production of natural products in bacterial systems, such as temperature, codon usage, and plasmid copy number [62,63,64]. Thus, optimizing these parameters will be examined.

Our results illustrated the in vivo functional expression of AtaPT and CstDXS in the *E. coli* system, which could be seen from the formation of ferulenol (2) and its isomeric structure (3) secreted into the culture medium. These findings also shed new light on the use of the engineered *E. coli* system as the prenylation system to produce valuable secondary metabolites instead of isolation from plants and chemical synthesis. Since the AtaPT used in this study exhibits broad substrate specificity towards many types of aromatic acceptors, e.g., benzophenones, xanthones, stilbenes, and chalcones [46], the future impact of clones carrying pCDFDuet-AtaPT-CstDXS on synthesizing the prenylated products might not be restricted to 4HC (1) but could be applied to the other types of aromatic acceptors.

## 4. Materials and Methods

### 4.1. Reagents

The general reagents were analytical grade and were purchased from Sigma-Adrich (St. Louis, MO, USA), Merck (Darmstadt, Germany), and Avantor (Center Valley, PA, USA).

### 4.2. Construction of Plasmid pCDFDuet-AtaPT-CstDXS

pCDFDuet-1 coexpression vector (Novagen, Darmstadt, Germany) was chosen to construct the recombinant plasmid containing the two genes encoding AtaPT (GenBank accession no. KP893683) and CstDXS (GenBank accession no. AB354578.1) based on the procedure reported by Toila and Joshua-Tor [65]. The initial insertion was performed by incorporating AtaPT in the MCS-1 (*BamHI* and *NotI*) followed by insertion of CstDXS into the MCS-2 (*Kpn*I and *Xho*I) of the pCDFDuet-1 vector to yield pCDFDuet-AtaPT-CstDXS. The details were as follows: The plasmid pUC57-AtaPT obtained from the gene synthesis technology (Invitrogen, Waltham, MA, USA) was used as the DNA template to provide AtaPT. The PCR reaction consisted of Phusion High-Fidelity DNA polymerase (NEB, Ipswich, MA, USA); the forward primer was AtaPT-F: 5′-GGTGGATCCGATGCTCCCCCCATCAGACA-3′, and the reverse primer was AtaPT-R 5′-AAAGCGGCCGTCACACAGCTGCG-3′ (underlines are the recognition sites for *BamH*I and *Not*I, respectively). The PCR cycle included pre-denaturation at 98 °C for 1 min, followed by 30 cycles of 98 °C for 30 s, 60 °C for 30 s, and 72 °C for 30 s, and a final extension at 72 °C for 5 min. The obtained PCR product (~1275 bp) was purified by using a Gel Band Purification Kit (GE Healthcare, Chicago, IL, USA), digested with *BamH*I and *Not*I, and ligated into a pCDFDuet-1 vector which had been treated with the same restriction enzymes. The ligation mixture was transformed into *E*. *coli* DH5α and the positive clones were selected by spreading on the LB-agar-contained streptomycin (50 μg/mL). The resulting plasmids were extracted using PureYieldTM Plasmid Mini-prep System (Promega, Madison, WI, USA) and the gene insertion was confirmed by double digestion with *BamH*I and *Not*I. The resulting pCDFDuet-AtaPT was used as the DNA backbone in the next step.

Based on the previous finding, the gene-encoded 1-deoxy-D-xylose 5-phosphate synthase of *C*. *stellatopilosus* (CsDXS: 2163 bp) was predicted to contain the putative chloroplast transit peptide (cTP) that should be removed before the gene expression in *E*. *coli* systems [40]. Therefore, the truncated CsDXS (CstDXS) which was absent of the cTP coding sequence (171 bp) was cloned from the young leaves of *C*. *stellatopilosus*. The PCR reaction consisted of Pfu DNA Polymerase (ThermoScientific, Waltham, MA, USA), CstDXS-F: 5′-TGCGGTACCATGGCATCACTTTCAGAAA-3′, and CstDXS -R: 5′-AGCCTCGAGTGCTGACATAATTTGCAGA -3′ (underlines are the restriction sites for *Kpn*I and *Xho*I, respectively). The PCR condition was as follows: Pre-denaturation at 95 °C for 1 min, followed by 30 cycles of 95 °C for 30 s, 60 °C for 30 s, and 72 °C for 2 min, and a final extension at 72 °C for 5 min. The PCR product (1992 bp) was purified from the agarose gel by using the gel purification kit, double digested with *Kpn*I and *Xho*I, and was ligated to the pCDFDuet-AtaPT which was digested with the same restriction enzymes. The double digestion by *Kpn*I and *Xho*I was performed to confirm the insertion of CstDXS in pCDFDuet-AtaPT (Figure S3). The nucleotide sequencing (IDT, Penang, Malaysia) was performed to verify the correct bases and the in-frame arrangement of both AtaPT and CstDXS in the pCDFDuet-AtaPT-CstDXS by using two pairs of primers: ACYCDuetUP1 primer 5′-GGATCTCGACGCTCTCCT-3′, and DuetDOWN1 primer 5′-GATTATGCGGCCGTGTACAA-3′ for AtaPT in the MCS-1, and DuetUP2 primer 5′-TTGTACACGGCCGCATAATC-3′ and T7term primer 5′-GCTAGTTATTGCTCAGCGG-3′ for CstDXS in the MCS-2.

### 4.3. Bioconversion of 4HC (1) into the Farnesylated-4HC Derivatives

The pCDFDuet-AtaPT-CstDXS transformed into *E*. *coli* BL21(DE3)pLysS (Promega, Madison, WI, USA) by heat shock method. The engineered strains carrying pCDFDuet-AtaPT-CstDXS were cultured in the LB medium containing streptomycin (50 μg/mL) and chloramphenicol (34 μg/mL) at 37 °C, 200 rpm for 18 h. The 1.5 mL of culture was then inoculated into the 500 mL Erlenmeyer flask containing 150 mL of the same medium and was further cultivated at 37 °C (200 rpm) until the OD_600_ reached 1.0. The gene expression was induced by adding 1 mM IPTG (final concentration); after that, cells were grown at 18 °C, 250 rpm for 5 h. To start the bioconversion process, 3 mM 4HC (1) (TCI, Tokyo, Japan) and 3 mM MgCl_2_ were supplied to the induced culture and the cells were further cultivated at the same condition for 18 h. After that, the culture medium was harvested by centrifugation at 4 °C, 8000 rpm for 10 min. *E*. *coli* BL21(DE3)pLysS carried pCDFDuet-AtaPT-CstDXS that was grown in parallel at the same condition, except without the supplement of 4HC (1) used as the control in this study. Since ferulenol (2) is light-sensitive, the bioconversion experiment throughout this study took place in the darkness to minimize the product degradation.

### 4.4. Extraction of Prenylated Products from the Culture Medium

The extraction process was performed in the darkness. The culture medium (150 mL) was partitioned twice with 75 mL EtOAc in the 500 mL Erlenmeyer flask by shaking at 300 rpm, 25 °C for 30 min. The EtOAc layers were then harvested after centrifugation for 5 min, 6000 rpm, at 4 °C, and concentrated until dryness using N_2_ gas. The dried residues were redissolved in MeOH (HPLC grade) before identification of the prenylated-4HC derivatives by using the high-resolution LC-ESI-QTOF-MS/MS in both negative- and positive-ion mode operations.

### 4.5. Identification of Metabolites by LC-ESI-QTOF-MS/MS

Identification of the prenylated products were carried out by HPLC Dionex (Thermo Scientific) connected with MS Bruker Maxis (esquire 4000 Daltonics, Bremen, Germany) and the RP-18 column (Acclaim RSCL 120 C18 column, 2.1 X 100 mm, 2.2 μM, Thermo Fisher Scientific, Waltham, MA, USA). The mobile phases consisted of H_2_O with 0.1% formic acid (solvent A) and acetonitrile with 0.1% formic acid (solvent B). The separation of prenylated products was achieved by using a linear gradient of solvent B as follows: 5% for 0–2 min, 5–95% for 15 min, 95% for 3 min, and back to 5% for 10 min, for a total running time of 30 min. The flow rate was set at 0.4 mL/min. The sample temperature was controlled to 10 °C. The column oven temperature was 40 °C. The injection volume was 10 µL. The product elucidation was conducted by tandem mass (MS/MS) with electrospray ionization (ESI) under the collision-induced dissociation (CID) energy 35 eV in negative ion mode analysis. Nebulizer pressure was set at 29 psi, the dry gas temperature was 180 °C, and the dry gas flow rate was 8.0 L/min. The masses were scanned over the *m/z* range of 100–1000 amu. The obtained MS/MS spectra (Figure S4) were directly compared against MS/MS spectra of ferulenol (2) [54], before being annotated by using the in silico tools to gain insight into the structure to ultimately confirm the obtained results. For positive ESI mode, the same chromatographic separation condition was applied for identifying both prenylated products (compound- 1 and 2), except the ion polarity was switched to the positive-ion mode with the CID energy of 25 eV (mass scan range 50–1500 amu). The obtained MS/MS spectra were then compared to the authentic ferulenol (2) (final concentration of 20 ppm) (Santa Cruz Biotechnology, Santa Cruz, CA, USA) run in parallel under the same positive ESI condition.

### 4.6. Structural Annotation of Prenylated Products by Using MetFrag

MetFrag, the freely accessible software (https://msbi.ipb-halle.de/MetFragBeta/ (accessed on 26 September 2021)), was used as a tool for structural annotation of metabolites from the high-quality MS/MS spectra of the query subjects, which plays a crucial role as the initial step of structural identification. According to the user manual provided in https://ipb-halle.github.io/MetFrag/projects/metfragweb/ (accessed on 26 September 2021), structural annotation of the target metabolites is required for two steps of data processing, including “retrieving candidates” and “processing of candidates”. Based on the experimental mass spectra of the compound-1, the neutral mass of 366.219495 (relative mass deviation of 5 ppm) with the calculated molecular ion (*m/z* 365.211670 [M − H]^−^) was defined as parameters to retrieve the candidate molecules from the PubChem database. The neutral molecular formula (C_24_H_30_O_3_) and data-specific identifiers (i.e., compound ID number) could also be defined in this step to perform the candidate search. Having retrieved a large number of candidates (2267 molecular structures, in our case) from PubChem, several parameters including the relative mass deviation (10 ppm), absolute mass deviation (0.001), the certain adduct type in ([M − H]^−^), and the MS/MS peak list (Figure S4) were then defined to match against “in silico generated fragments” of candidates from the PubChem database. After that, the score-ranked list of candidates was displayed. For each candidate in the row, the information regarding candidate image, identifier with linked database, exact mass, molecular formula, and several MS/MS peaks explained were also displayed. For a certain candidate of the list, “fragment view” also provided molecular formulas and their corresponding substructures for the matched peaks, which are highlighted in green.

### 4.7. Structural Annotations of Prenylated 4HC Analog Using SIRIUS Tool

SIRIUS is an in silico tool developed for the identification of molecular formula, mass spectra, and fragmentation tree annotation of the unknown metabolites based upon high-resolution raw MS/MS data [54]. This tool is also connected with CSI:FingerID web service for structural identification and prediction substructures (so-called molecular fingerprints) present in the query subject. SIRIUS can also systematically predict the classes of query metabolites via the computational tool (CANOPUS: class assignment and ontology prediction using mass spectrometry) [56]. Structural elucidation of the prenylated-4HC analogs were thus based on the prediction power of SIRIUS (version 4.8.2). According to the user manual, the raw MS/MS data in text format (Figure S4) was imported into the SIRIUS application window. The MS2 level and the collision energy (35 eV) were subsequently defined in the next dialogue. Then, two parameters, including the precursor mass ion (*m/z* 365.2154) and the specific adduct type ([M − H]^−^), were defined in the following application window. The annotation of prenylated-4HC derivatives was accomplished by selecting “compute option”. In our case, the entire tools, including SIRIUS, CSI:FingerID web service, and CANOPUS, were selected to sufficiently detail structural elucidation of the prenylated product. Certain databases (e.g., Natural Products, COCONUTS, and NORMAN) were also chosen as biological databases to improve the rate of correct identification and rank of the query metabolite.

## 5. Conclusions

In this study, we demonstrated that the *E coli* BL21(DE3)pLysS strain could be engineered as the prenylation system of 4HC (1) via co-expression of the genes encoding for AtaPT and CstDXS. Based on the high-resolution LC-ESI(-)-QTOF-MS/MS, feeding 4HC (1) into the engineered strain resulted in the formation of the prenylated 4HC analog (namely compound-1) showing *m/z* 365.21 [M − H]^−^, which was detected from the culture medium. The mass fragmentation pattern of this product was highly identical to the authentic ferulenol (2) established by the previous work [66]. MetFrag analysis clearly showed that the compound was best explained as “ferulenol (2)” among 2267 candidated compounds from the PubChem database. Further annotation using SIRIUS integrated with CSI:FingerID and CANOPUS led to unraveling the chemical features hidden in compound-1, e.g., molecular formula (defined as C_24_H_30_O_3_), ferulenol (2) as a potential structure, and classes (defined as coumarin derivatives), indicating the prenylate-4HC derivative was indeed ferulenol (2). Meanwhile, compound-2 (*m/z* 365.21 [M − H]^−^ and 367.22 [M + H]^+^) was elucidated as the farnesylated of 2-hydroxy-4-chromenone (c), which was proposed to be formed through the tautomerization of ferulenol (2). It is worthwhile to emphasize that both products could be formed without the external supply of prenyl donors (e.g., DMAPP, GPP, and FPP), which indicated the capacity of *E*. *coli* to supply the prenyl donors, particularly FPP, and verified the functional expression of AtaPT and CstDXS in this bacterial system. Equally important, they were secreted in the culture medium, providing a great benefit from an economic point of view since breaking cell pellets is not needed. Further experiments, e.g., overexpression of rate-limiting step enzymes in the DXP pathway along with the optimized culture conditions, are required to improve the yields of two products. Based on the substrate promiscuity of AtaPT, the in vivo prenylation by clones carrying pCDFDuet-AtaPT-CstDXS could be applied towards other types of natural products such as quercetin and mangiferin to generate diverse classes of prenylated derivatives.

## Supplementary Materials

The following are available online, Figure S1: The insertion of AtaPT in MCS-1 of pCDFDuet-1 vector, Figure S2: The insertion of CstDXS in MCS-2 of pCDFDuet-1 vector, Figure S3: The verified insertion of CstDXS (~1992 bp) in pCDFDuet-AtaPT (~5020 bp) via *Kpn*I and *Xho*I digestion, Figure S4: The raw mass (MS2) data for putative ferulenol (2) (compound-1) obtained from negative-ESI mode, Figure S5: The proposed reaction mechanisms illustrating the various chemical losses present in the MS/MS spectrum of compound-1, Figure S6: The proposed reaction mechanisms illustrating the various chemical losses present in the MS/MS spectrum of compound-2, Figure S7: The postulated mechanisms underlying the formation of ion *m**/z* 189.05 of compound-1, Figure S8: The postulated mechanisms underlying the formation of ion *m**/z* 189.05 of compound-2.

## Sample Availability

Samples of the compounds are not available from the authors.
