# Supplementary material for: Synthesis of Ferulenol by Engineered Escherichia coli: Structural Elucidation by Using the In Silico Tools
Source: Molecules. 2021 Oct 16;26(20):6264. doi: 10.3390/molecules26206264 (PMC8537342; doi:10.3390/molecules26206264)

Supplementary Materials

Figure S1: The insertion of AtaPT in MCS-1 of pCDFDuet-1 vector

CGGGAATCGAATCGCGAGTATACGACTCACTATAGGGGAATTGTGAGCGGATAACAATCCC

RBS **6XHIS**

CCTCTAGAAATAATTTTGTTTAACTTTAAG**AAGGA**GATATACCATGGGCAGCAGC**CATCAC**

6XHIS ***Bam*HI** **Start codon**

CATCATCACCACAGCCAGGATCCG**ATG**CTCCCCCCATCAGACAGCAAAGATCCCCGGCCCT

GGCAGATCCTGAGCCAGGCGCTCGGCTTCCCCAATTACGACCAGGAACTGTGGTGGCAAAA

CACGGCGGAGACGCTCAACCGCGTCCTCGAACAGTGCGATTACAGCGTGCATCTGCAGTAC

AAGTACCTCGCCTTCTACCACAAATACATCCTCCCCAGTCTGGGCCCCTTCCGCCGCCCGG

GCGTCGAGCCCGAGTACATCAGCGGCCTCTCCCACGGCGGCCATCCGCTGGAGATCAGCG

TCAAGATCGACAAGTCCAAAACGATCTGCCGGCTCGGATTGCAGGCGATCGGGCCGCTCGC

CGGCACCGCGCGCGACCCGCTCAACAGCTTCGGCGACCGTGAACTCCTCAAGAATCTCGCG

ACGCTGCTGCCGCACGTCGACCTGCGCTTGTTCGACCACTTCAACGCGCAGGTCGGGCTCG

ACCGCGCGCAGTGCGCCGTCGCCACCACCAAGCTCATCAAGGAGAGCCACAACATCGTCTG

CACGTCGCTCGACCTCAAAGACGGCGAGGTCATTCCCAAGGTCTACTTCTCCACCATCCCG

AAGGGGCTCGTCACCGAAACGCCGCTGTTCGACCTCACCTTTGCGGCGATCGAGCAGATGG

AGGTATACCACAAAGACGCGCCGCTGCGGACGGCGCTGTCGTCGCTGAAGGACTTTTTGCG

CCCGCGCGTGCCCACCGACGCCAGCATTACCCCGCCCCTGACCGGCCTCATCGGCGTCGAT

TGTATCGACCCCATGCTGTCCCGGCTGAAGGTGTACCTGGCCACCTTTCGCATGGACCTGT

CCTTGATCCGCGACTACTGGACGCTGGGCGGCTTGCTGACGGACGCGGGCACGATGAAAGG

CCTGGAGATGGTGGAGACGCTGGCTAAGACGCTGAAGCTGGGCGACGAGGCCTGCGAGACG

CTGGACGCGGAGCGTCTGCCGTTTGGAATCAACTATGCGATGAAGCCGGGGACGGCGGAGT

TGGCGCCGCCCCAGATCTACTTCCCGCTGCTGGGCATTAATGACGGGTTCATTGCGGATGC

TCTAGTTGAATTCTTCCAGTACATGGGCTGGGAGGACCAGGCGAATCGGTACAAGGATGAA

CTGAAGGCGAAATTTCCCAATGTGGATATCTCGCAGACCAAGAACGTCCATCGCTGGCTGG

GAGTGGCGTACTCCGAGACCAAGGGGCCGTCGATGAACATTTACTACGATGTGGTTGCGGG

Stop codon

AAATGTCGCACGTGTG**TGA**GCGGCCGC

***Not*I**

Figure S2: The insertion of CstDXS in MCS-2 of pCDFDuet-1 vector

RBS

TCTTAGTATATTAGTTAAGTATAAG**AAGGAG**ATATACATATGGCAGATCTCAATTGGATAT

*Kpn*I **Start codon**

CGGCCGGCCAGCCGATCGCTGACGTCGGTACC**ATG**GCATCACTTTCAGAAAGTGCAGAGTA
TCACTCAAACAGACCAGCAACACCTCTACTGGACACCATCAACTTTCCAATTCATATGAAA
AATCTATCAATCAAGGAACTGAAGCAGCTAGCAGAAGAGCTCCGTTCAGATGTTATTTTCA
ATGTTTCTAAAACTGGGGGTCACTTGGGATCAAGCCTTGGTGTTGTTGAACTTACTGTGGC
TCTTCACTATATTTTCAATACTCCTCAAGATAAGATTCTGTGGGATGTTGGCCATCAGTCT
TACCCCCACAAAATCCTTACCGGGAGAAGAGACAGGATGCGCACAATTAGACAGACTAATG
GGCTTTCTGGGTTCACCAAACGAGCTGAGAGCGAACATGATTGCTTTGGCACTGGCCATAG
CTCTACCACTATTTCTGCAGGCTTGGGAATGGCAGTGGGGAGAGATTTAAAAGAAAGAAAG
AACAATGTTGTGGCTGTTATAGGAGACGGAGCCATGACAGCAGGACAAGCTTATGAAGCTA
TGAACAATGCAGGATACTTGGACTCCGATATGATTGTTATTCTCAATGACAACAAACAGGT
TTCTTTACCAACTGCTACGCTTGATGGACCAATACCACCTGTGGGAGCTTTGAGCAGTGCT
CTTAGCAGGTTGCAATCCAACCGGCCTTTAAGAGAACTAAGAGAGATTGCCAAGGGTGTTA
CAAAGCAAATTGGTGGACAGATGCATGAGTTGGCTGCAAAGGTTGATGAATATGCACGTGG
TATGATAAGTGCTTCTGGATCATCTCTTTTTGAAGAGCTTGGTTTGTATTATATTGGTCCA
GTTGACGGTCACAATATCGAGGATCTTGTCGATATCCTTAAAGAGGTTAAAAGCACCAAA
TCAACTGGGCCAGTCTTGATTCATGTCGTTACTGAGAAAGGCCGGGGATATCCATATGCTG
AGAGAGCCGCAGACAAGTACCACGGAGTAACCAAGTTTGATCCTGCAACCGGGAAGCAATT
TAAGTCTGGTGGAAGTACACAGTCGTACACGACATACTTTGCAGAGGCTTTGATTGCAGAA
GCAGAAGTGGACAAGGATATTGTCGCAATTCATGCTGCAATGGGAGGAGGAACAGGCTTGA
ATCTCTTCCATCGCCGTTTCCCAAATAAATGCTTTGATGTTGGGATAGCAGAGCAGCATGC
TGTTACATTTGCTGCTGGATTGGCCTGCGAAGGCCTTAAACCTTTCTGTGCTATCTATTCA
TCTTTCATGCAGAGGGCTTACGACCAGGTGGTGCACGATGTGGATTTGCAGAAACTTCCTG
TAAGATTCGCGATGGATAGAGCTGGCCTCATCGGAGCAGATGGTCCCACACATTGTGGAGC
CTTTGATGTTACTTTCATGGCGTGCCCTCCTAACATGGTGGTGATGGCTCCTTCTAATGAA
GCAGAACTCTTTAACATGGTTGCCACTGCTGCTGCCATAGATGATCGTCCAAGCTGTTTCC
GATATCCGAGAGGAAACGGTATCGGTGTTCCNCTGCCNCCTGGAAACAAAGGCATTCCTNT
TGAGGTTGGAAAAGGAAGGATCTTGATTGAGGGGGAGCGAGTGGCGCTACTGGGATATGGT
GCAGCAGTTCAAAACTGTCTAGCTGCTGCATCTTTAGTTGAAACCCCCGGCTTACGTGTAA
CTGTTGCAGATGCTAGATTTTGTAAACCATTAGATCAATCTCTGATTCGAAGTCTAGCGAA
GTCNCACGAAGTTTTGATTACGGTTGAAGAAGGCTCAATTGGGGGATTTGGATCTCATGTT
GCTCATTTTATGGCTCTCAATGGCCTTCTTGATGGCANCCTTAAGTGGCGACCGCTCGTTN
TTCCCGATCGATATATTGAGCNCGGATCNCCGGCAGACCAGTTGATAGAGGCAGGTTTAAC
NCCATCTCNCATTGCCGCAACAATATTCAACATACTTGGAAACAAAAGAGAAGCTCTGCAA
ATTATGTCAGCACTCGAGTCTGGT**AAAGAAACCGCTGCTGCGAAATTTGAACGCCAGCACA**
***Xho*I** **S.Tag**
TGGACTCG

Figure S3: Verified insertion of CstDXS (~1992 bp) in pCDFDuet-AtaPT (~5,020 bp) via *Kpn*I and *Xho*I digestion

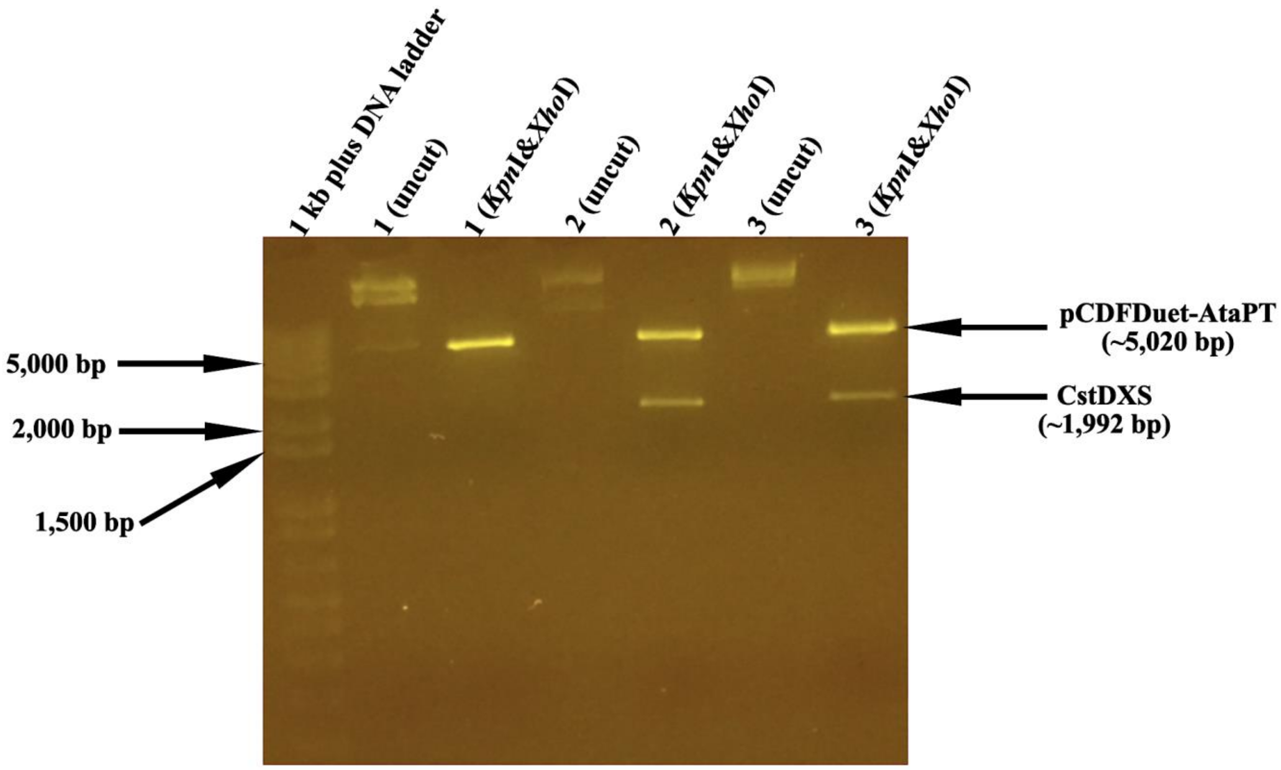

Figure S4: The raw mass data for putative ferulenol (2)

Compound-1<br>Molecular ion 365.2120 RT 21.3 min	
**m/z**	**Intensity**
96.9611	128
120.0216	132
161.0249	400
174.0305	436
187.0397	580
200.0827	172
213.0559	656
214.0597	308
227.0714	604
228.02792	1432
229.02792	416
365.2154	960

Figure S5: The proposed reaction mechanisms illustrating the various chemical losses present in the MS/MS spectrum of compound-1

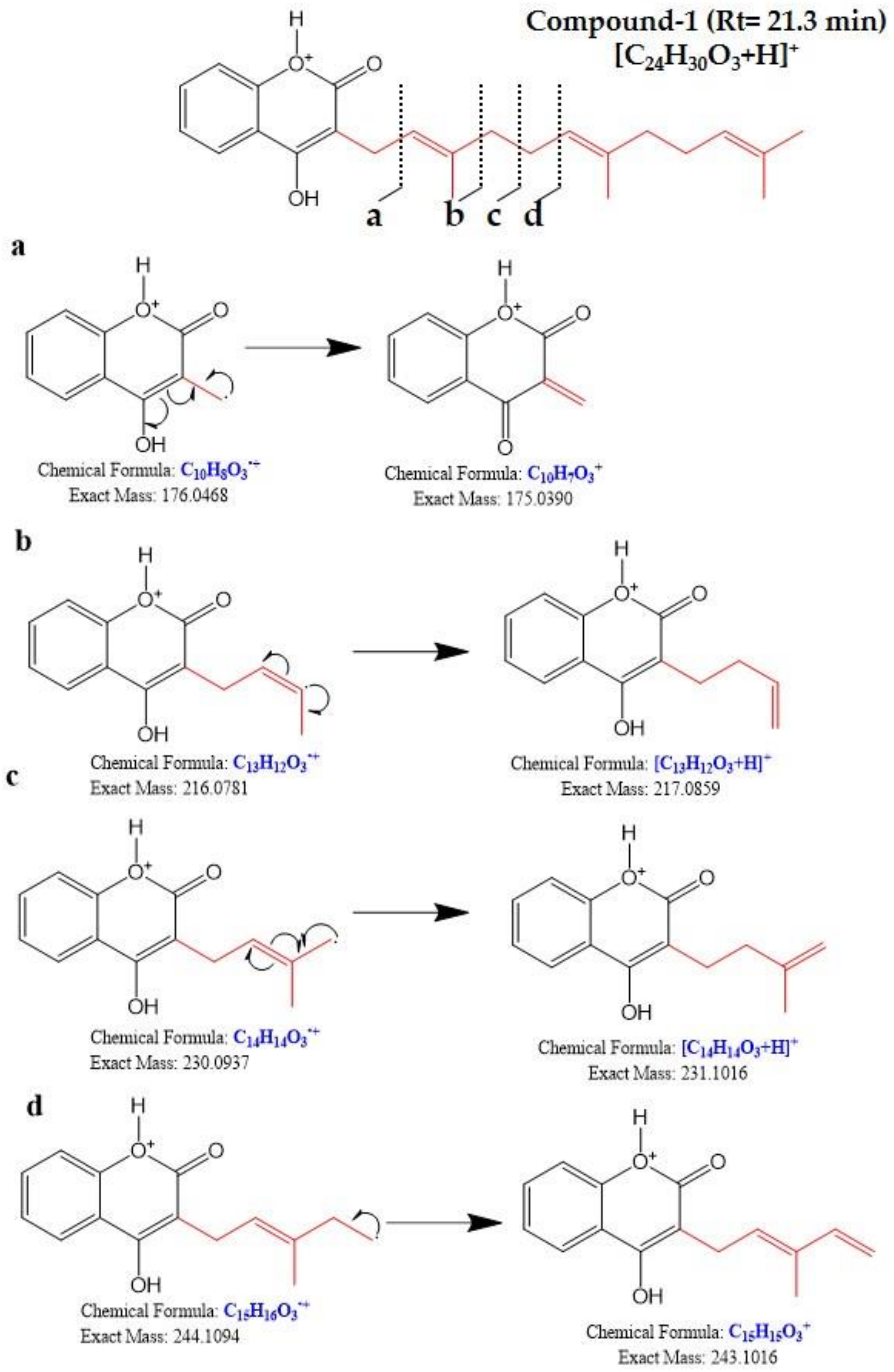

Figure S6: The proposed reaction mechanisms illustrating the various chemical losses present in the MS/MS spectrum of compound-2

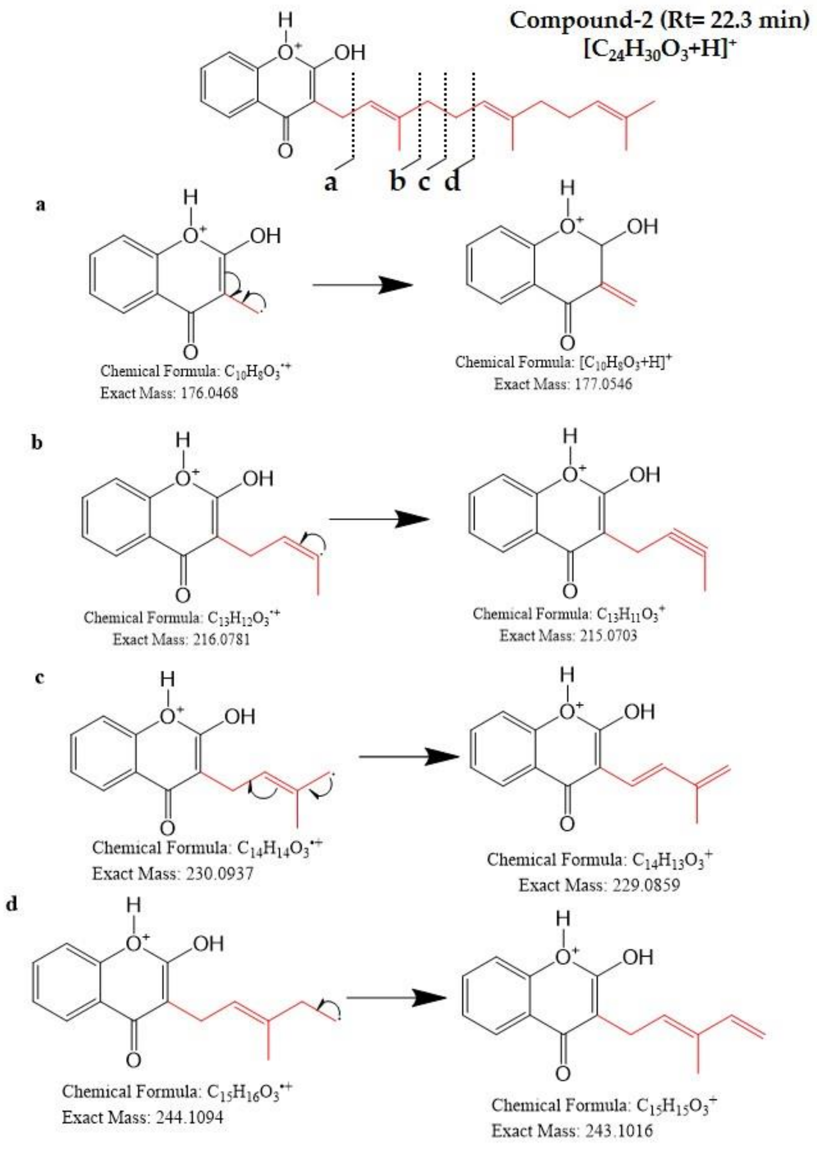

Figure S7: Figure S7: The postulated mechanisms underlying the formation of ion *m/z* 189.05 of compound-1

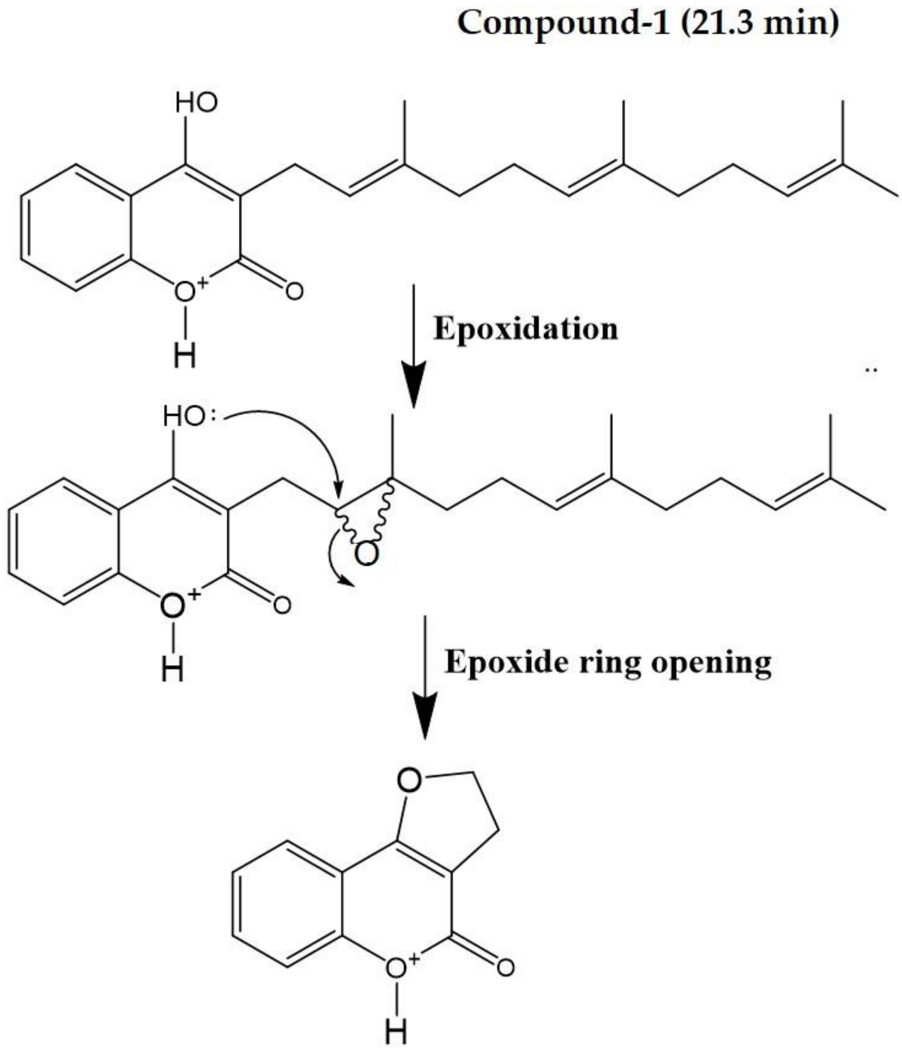

$C_{11}H_9O_3^+$

m/z **189.05**

Figure S8 The postulated mechanisms underlying the formation of ion *m/z* 189.05 of compound-2

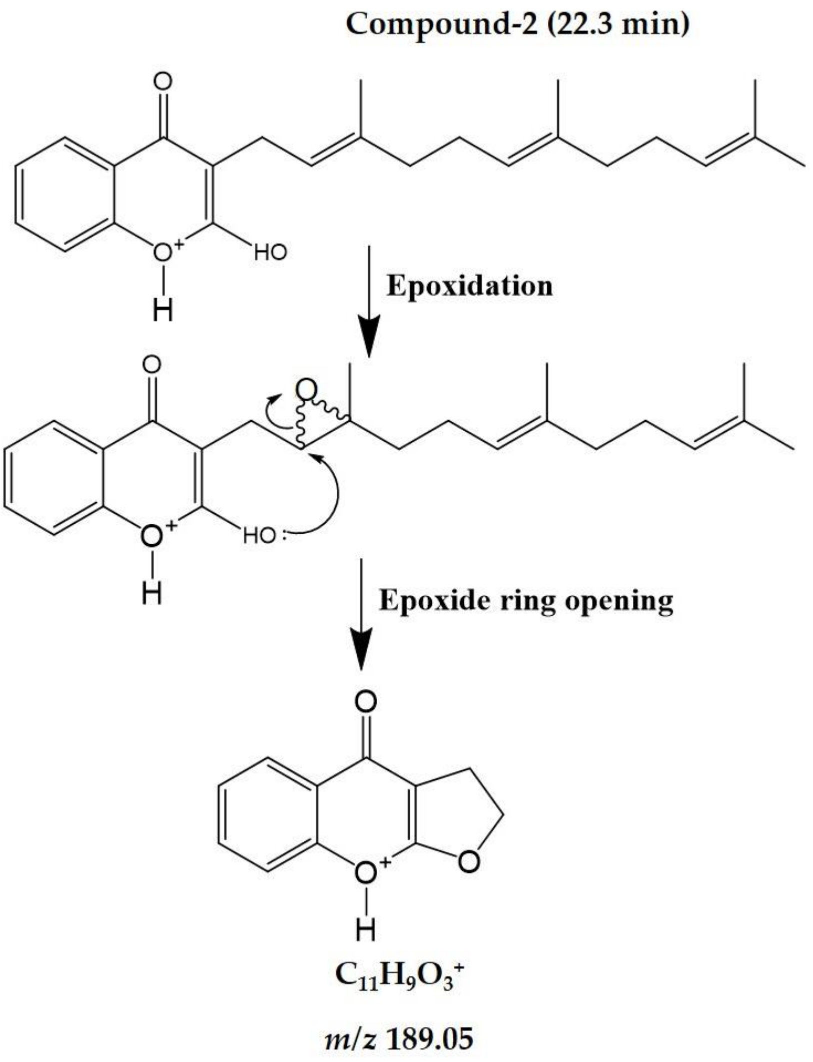

Supplement: Supplementary file 1 [file molecules-26-06264-s001.zip › molecules-1315641-supplementary.pdf]
